# Leading public health innovation in high schools across local and global settings

**DOI:** 10.3389/fpubh.2026.1733013

**Published:** 2026-04-08

**Authors:** Ashish Joshi, Niharika Jha, Kami Geron, Michelle Jeu, Laura Granack, Stephen Shular, Manoj Kumar, Tejinder Kaur, Praveen Guleria, Sanjeev Kumar, Neville Calleja, Lori Ward, Ashraf Elmetwally, Noof Alwatban, Megha Bhatnagar, Rafaela Rosário

**Affiliations:** 1 School of Public Health, University of Memphis, Memphis, TN, United States; 2 DAV University, Jalandhar, India; 3 Department of Public Health, Faculty of Medicine and Surgery, University of Malta, Msida, Malta; 4 College of Public Health and Health Informatics, King Saud bin Abdulaziz University for Health Sciences, Riyadh, Saudi Arabia; 5 King Abdullah International Medical Research Center (KAIMARC), Riyadh, Saudi Arabia; 6 SDRV Convent School, Greater Noida, India; 7 School of Nursing, University of Minho, Campus of Gualtar, Braga, Portugal

**Keywords:** education, experiential learning, innovation, public health, public health in action

## Abstract

**Introduction:**

Adolescents in the 21st century face numerous preventable public health challenges that extend beyond academics to include physical, mental, and social well-being. Addressing these issues requires schools to integrate public health education into their systems, empowering youth with digital health literacy and interdisciplinary skills such as critical thinking, problem-solving, communication, innovation, and collaboration. To meet this need, the University of Memphis School of Public Health developed the “RE-AIM Public Health IDEAS” model (Research, Entrepreneurship, Analytics, Informatics, and Management), designed to transform innovative ideas into actionable solutions.

**Methods:**

Complementing this framework, the initiative promotes Leadership and Educational Advancement among Youth to solve Public Health (LEAP) challenges, enabling students to gain exposure to population-level thinking and community health problem-solving. The model is operationalized through the *Public Health in Action* curriculum, an innovative, evidence-informed, and flexible approach that combines foundational training in public health with real-world experiential learning. Using principles of human-centered design and data-driven strategies, the curriculum emphasizes critical skills in science and digital health literacy, youth empowerment, and community-based participatory action research. Implementation details were presented through descriptive summaries, supplemented by graphs and tables illustrating student participation and activity outcomes.

**Results:**

Public Health Clubs established in high schools across the United States and India serve as structured platforms where students engage in collaborative learning, team-building, and creative activities while addressing pressing health issues in their communities. A total of 400 students contributed to approximately 150 health-focused activities aligned with the Sustainable Development Goals, including hygiene promotion, nutrition awareness, mental well-being, and preventive healthcare. By familiarizing youth with health information, organizational skills, and leadership opportunities, the program fosters social responsibility and normalizes positive health behaviors that can have lasting effects on both personal and community health outcomes.

**Conclusion:**

This initiative inspires young people to pursue careers in public health, enhances sensitivity to health issues, and cultivates the next generation of public health ambassadors dedicated to translating education into meaningful, community-driven impact.

## Introduction

Adolescents today face an expanding spectrum of public health challenges, including mental health disorders, vaping and substance misuse, cyberbullying, rising non-communicable disease (NCD) risks, and climate-related health threats. Evidence consistently shows that unhealthy dietary patterns, excessive screen time, and physical inactivity have driven childhood obesity to alarming levels worldwide (1, 2). Globally, approximately one in seven adolescents aged 10–19 years experiences a mental disorder, making mental health conditions a major contributor to disease burden in this age group (3). Suicide has also emerged as a critical concern and is now the third leading cause of death among young people aged 15–29 years worldwide (3). At the same time, lifestyle-related risk factors are rapidly increasing. Evidence indicates that unhealthy diets, sedentary lifestyles, and excessive screen time have contributed to a global rise in childhood obesity, with projections suggesting that nearly 464 million adolescents may be overweight or obese by 2030 (4). These trends highlight the growing intersection between behavioral, environmental, and social determinants of adolescent health.

Although these risk factors are largely preventable, current public health and educational systems often fail to provide adolescents with the skills and environments needed to make informed health decisions. Schools, where young people spend most of their waking hours, remain one of the most influential settings for shaping health knowledge, attitudes, and behaviors. Empirical research underscores that school-based health programs can improve academic achievement, enhance mental and social well-being, and cultivate essential 21st-century competencies such as communication, critical thinking, collaboration, and problem-solving (5, 6).

Adolescence represents a critical developmental period characterized by profound physical, cognitive, and emotional changes that shape long-term health behaviors and life trajectories. During this stage, young people begin forming attitudes, habits, and decision-making patterns that often persist into adulthood. Effective health education during adolescence can therefore play a pivotal role in strengthening non-cognitive competencies, improving health-related quality of life, and narrowing the gap between life aspirations and life satisfaction (7–10). In an increasingly complex global environment, both public and private sectors are placing greater emphasis on a future workforce equipped not only with academic knowledge but also with 21st-century skills such as critical thinking, collaboration, adaptability, and responsible decision-makin (9). Integrating public health perspectives into secondary education can help prepare students to navigate health challenges while simultaneously developing the competencies needed for lifelong well-being and productive participation in society.

To support these developmental processes, theory-driven models can help explain how adolescents absorb and transform information into meaningful actions and innovations. For example, the Granular Interaction Thinking Theory (GITT) (11) which proposes that individuals process knowledge through interactions with small units of information, often referred to as “information granules.” These granules originate from multiple sources, including formal education, peer interactions, personal experiences, and digital environments. According to GITT, individuals do not passively receive information; rather, they actively filter, combine, and reinterpret these granules through cognitive and social processes to produce mental outputs such as values, judgments, decisions, and creative ideas (11–13). In learning environments where diverse information sources are intentionally structured and connected, this process can stimulate deeper understanding, creativity, and problem-solving. Applying this perspective to adolescent education suggests that schools can play a critical role in shaping how students engage with information and translate it into innovative responses to real-world challenges. In this way, theory-informed educational models can strengthen students’ capacity to generate creative and contextually relevant solutions to public health problems.

Despite increasing recognition of the importance of adolescent health and youth engagement, there remains a notable research gap: few school-based initiatives systematically integrate public health education, innovation training, and real-world application through a theoretical model. Existing programs typically focus on narrow health topics or short-term activities, lacking a comprehensive, theory-driven framework that equips adolescents to identify local problems and develop feasible solutions. This gap highlights the urgency of designing models that not only educate but also empower youth to become active contributors to community health. To address this need, the Re-AIM Public Health IDEAS initiative was intentionally designed with a primary focus on improving adolescent health literacy and awareness through applied, experiential learning, while secondarily fostering foundational public health competencies appropriate for high school students (14). These competencies include basic skills such as recognizing common community health challenges, interpreting health information, understanding prevention strategies, and communicating health messages effectively to peers and family members. The initiative does not seek to train students as public health professionals; rather, it aims to equip youth with the knowledge, confidence, and practical skills needed to recognize public health issues, make informed health decisions, and participate constructively in community health improvement efforts.

The *Re-AIM Public Health IDEAS* initiative, developed by the University of Memphis School of Public Health, addresses this gap by introducing an innovative framework for establishing public health clubs alongside a hands-on curriculum, *Public Health in Action* (14). The initiative aims to equip students with the capacity to generate creative, evidence-informed solutions to public health challenges across local and global contexts.

Therefore, the objective of this study is to describe an innovative, first of its kind initiative “Re-AIM Public Health IDEAS” through the lens of the youth as a model of establishing public health clubs in the high schools by implementing a unique curriculum Public Health In Action helps students with the essential skills and knowledge to come up with out-of-the-box solutions to address common public health challenges in local and global settings.

## Methods

This study employed a descriptive case study approach to document the development and implementation of the Re-AIM Public Health IDEAS initiative across participating high schools. The purpose of this approach was to provide a detailed account of how the model was designed, introduced, and operationalized within real school settings. Specifically, the study describes the structure of the initiative, the establishment of Public Health Club activities, and the implementation of the Public Health in Action curriculum. This study focuses on the process of model development and implementation to illustrate how the initiative functioned in practice and how students engaged with its components.

Data were collected from multiple program components, including records of student activities, project focus areas, Public Health Club activities mapped to the Sustainable Development Goals (SDGs), club activities by school, and internal program documents detailing curriculum components and implementation processes. These data sources offered insights into program structure, student participation, and the types of public health issues addressed by students.

Data were summarized using descriptive narrative accounts, tables, and graphical presentations derived from program implementation records and activity documentation. The summarization process involved reviewing program materials, including student project titles, club activity reports, and curriculum implementation notes, and organizing this information into categories relevant to the study objectives, such as student project topics, alignment of Public Health Club activities with Sustainable Development Goals (SDGs), and levels of student engagement across schools. Frequencies and distributions were then presented through tables and figures to illustrate patterns across participating schools. Descriptive summaries were used to provide a transparent account of how the model functioned in practice.

### Ethical considerations

This manuscript reports on the implementation of the Re-AIM Public Health IDEAS initiative, a pilot school-based educational program conducted in collaboration with participating high schools. The initiative was implemented as an educational activity, and the present manuscript represents a retrospective descriptive analysis of routine program documentation and participation records.

Because participants were minors, authorization for student participation was obtained through established school approval processes. Responsibility for parental consent and student assent was managed by the participating schools in accordance with their internal policies for school-sponsored student activities. All program activities were conducted with the knowledge and approval of school administrators.

For the purposes of this manuscript, only de-identified program records and aggregated participation information were analyzed. No individual-level identifiers were collected or reported. Based on the retrospective use of de-identified program documentation and the absence of direct interaction with participants for research purposes, the activity was considered to fall outside the scope of human subjects research requiring full Institutional Review Board (IRB) oversight (15, 16). The study was conducted in accordance with institutional and ethical guidelines for responsible research, including the protection of confidentiality and accurate reporting of program activities.

### Findings

The *Re-AIM Public Health IDEAS* initiative resulted in the development of a structured model for integrating public health education and innovation training into high school settings. The initiative was implemented across participating high schools through a structured set of activities designed to introduce students to public health concepts and innovation-oriented learning. During the implementation period, schools established Public Health Clubs that served as the primary platform for student engagement (17). Through these clubs, students participated in structured sessions based on the *Public Health in Action* curriculum (14), which included discussions of community health challenges, collaborative project development, and student-led activities addressing locally relevant public health topics. Students worked in teams to identify health issues within their communities and developed project ideas aligned with selected Sustainable Development Goals (SDGs).

### Model of public health education in high schools

“RE-AIM Public Health IDEAS” through the Lens of the Youth is a University of Memphis School of Public Health Model for public health education in high schools (17). The initiative is built upon the relevance of transforming ideas into implementation for solving pressing public health challenges through Research, Entrepreneurship, Analytics, Informatics and Management (RE-AIM). The initiative aims to instill Leadership and Educational Advancement among youth to solve Public Health (LEAP) challenges of the 21st century. This initiative provides youth to *gain exposure to the skills of population thinking through public health education.* High school public health education focuses on critical thinking, science and digital health literacy, youth empowerment, and community-based participatory action research in each community. Youth will be empowered on how to acquire, analyze, evaluate, interpret, and apply information into daily life, allowing them to understand societal problems in a real-world context. It also helps to cultivate a sense of social responsibility to address community health needs.

The initiative is built on 5 pillars including (a) Coping, (b) Adaptability, (c) Resilience and (d) Empathy and Success (CARES). Students are facilitated by experts in the field to come up with solutions to address common public health challenges in local and global settings. The goal is to encourage creative and innovative thinking that will help translate ideas into designing and developing public health solutions to support community health and well-being. This platform brings together youth’s ideas on how today’s pressing public health problems can be addressed across diverse local and global settings. The model is implemented using an innovative, creative, data driven, evidence-informed curriculum for brining public health education into high schools through” Public health in Action” (18).

#### Public health in action curriculum

The curriculum aims to sensitize individuals about the importance of public health in a fast rapidly changing environment (17). The curriculum is implemented is implemented in collaboration with the International Education and Research Network (IEARN) and also as part of the Public Health Clubs established across various high schools locally and globally in Fall 2024 and Spring 2025 The curriculum is available as a series of interactive modules available through CANVAS platform and can be accessible at any point. Each module has a series of assessments and problem-solving exercises to assess students’ knowledge and competencies based on the learnings from the curriculum. For the public health club activities, there are in-person sessions that range from 1 to 2 h either during or after school, as desired by the school principals and based on the availability of the students and class schedules accordingly.

This unique curriculum blends foundational skills in public health combined with real world experience of addressing public health challenges impacting local and international communities through the lens of human centric principles and design thinking approaches for greater community impact. Individuals get oriented to the (a) field of public health, (b) its importance and relevance, role of data driven, evidence-informed research in public health, understanding of community needs, converting data into meaningful information and insightful knowledge, using human centric approaches to develop solutions that are Sustainable, Multisectoral, Accessible, Affordable, Reimbursable and Tailored (SMAART). Further students get exposure to storytelling with data, innovation and entrepreneurship, and using principles of design thinking develop public health interventions to enhance population health outcomes across diverse settings. The curriculum includes wide range of 14 topics (Table 1) that provide students with foundational knowledge of public health, explores the importance of research in public health, methods of data collection in public health, applying the principles of data, information and knowledge, storytelling with data, using human centered design to develop public health solutions, and methods of evaluation of the proposed interventions, discuss the role of new emerging technologies and its applications in public health, highlight the role of public health entrepreneurship in addressing public health challenges and how public health today is glocal with its intersections both at local and global levels. Further, there are 4 case studies that elaborate on the examples of public health interventions, innovations, programs and policies that address current and new public health challenges at the time of uncertainty, complexity and unpredictability in public health.

**Table 1:** Modules of public health in action curriculum.

Topic	Description	Learning objectives
Public Health 101	Serves as an introduction to public health, giving students a foundational understanding of key concepts, principles, and practices. Through lectures, discussions, and interactive activities, students will explore public health’s role in promoting and protecting population health.	Understand the definition and scope of public health. Explore the core functions and essential services of public health. Discuss the interdisciplinary nature of public health and its relationship to other fields.
Importance of research in public health	Focuses on the significance of research in advancing our understanding of health issues, informing evidence-based interventions, and improving population health outcomes. Through this module, students will explore the role of research in public health practice, its ethical considerations, and various research methodologies used in the field.	Understand the importance of research in informing public health policies, programs, and interventions. Explore the ethical principles and considerations in conducting research involving human subjects in public health. Examine different research methodologies used in public health, including quantitative, qualitative, and mixed methods approaches.
Data Gathering Techniques in Public Health	Introduces students to various methods and tools used for collecting, analyzing, and interpreting data in public health research and practice. Through this module, students will learn about the importance of data in identifying health trends, assessing population health needs, and monitoring the effectiveness of public health interventions.	Understand the importance of data gathering in public health for surveillance, monitoring, and evaluation purposes. Explore different data collection methods used in public health, including surveys, interviews, focus groups, and observational studies. Learn about data management techniques and storage to ensure data quality and integrity. Analyze the strengths and limitations of different data sources, such as primary and secondary data, in public health research and practice. Examine ethical considerations related to data collection, storage, and sharing in public health.
Story telling with public health data	Explores the art and science of using data to communicate compelling narratives about public health issues. Through this module, students will learn how to transform complex data into accessible and impactful stories that engage diverse audiences, raise awareness, and drive action towards improving population health.	Understand the power of storytelling in public health communication and advocacy. Learn techniques for effectively presenting public health data using visualizations, narratives, and multimedia tools. Explore different storytelling frameworks and approaches for conveying key messages about health disparities, disease outbreaks, and other public health challenges.
Role of Data, Information, and Knowledge in Public health	Explores the interplay between data, information, and knowledge in shaping public health policies, programs, and interventions. Through this module, students will examine how data is synthesized and transformed into actionable knowledge to address population health challenges.	Understand the concepts of data, information, and knowledge and their relevance to public health practice and research. Explore the data lifecycle in public health, including data collection, processing, analysis, interpretation, and dissemination. Examine the role of information systems and technologies in managing and sharing public health data effectively. Learn about knowledge dissemination strategies, including peer-reviewed publications, reports, and policy briefs. Analyze how knowledge generation and translation contribute to evidence-based decision-making in public health.
Human-Centered Design Approaches in Public Health	Teach students about innovative methods for developing and implementing public health interventions that prioritize the needs, preferences, and experiences of communities and individuals. Through this module, students will learn how to apply principles of human-centered design to address complex public health challenges	Understand the principles and methodologies of human-centered design and their relevance to public health practice. Examine the examples of human-centered design approaches applied to various public health issues
Evaluation of Public Health Intervention	Provides students with an understanding of evaluation methods and techniques used to assess the effectiveness, efficiency, and impact of public health programs and interventions. Through this module, students will learn how to design and conduct evaluations, interpret findings, and use evaluation results to inform decision-making and improve program outcomes.	Understand the importance of evaluation in assessing the effectiveness and impact of public health interventions. Learn about different types of evaluation approaches, including process evaluation, outcome evaluation, and impact evaluation.
Technological Innovations in Public Health	Explains the role of emerging technologies in transforming public health practice, research, and policy. Through this module, students will examine how advances in digital health, data analytics, mobile technologies, and artificial intelligence are revolutionizing disease surveillance, health promotion, healthcare delivery, and health outcomes monitoring.	Understand the potential of technological innovations to address public health challenges and improve population health outcomes. Explore key technologies and tools used in public health, such as electronic health records, telemedicine, and health apps. Examine various examples of successful implementation of technological innovations in public health programs and interventions.
Public Health Entrepreneurship	Explores the intersection of public health and entrepreneurship, focusing on the development, implementation, and sustainability of innovative solutions to address public health issues. Through this module, students will learn about the principles of entrepreneurship, business models, funding mechanisms, and partnerships in the context of public health.	Understand the concept of entrepreneurship and its application in the public health context. Explore the role of entrepreneurs, startups, and social enterprises in driving innovation and impact on public health. Learn about models and funding options for public health ventures. Discuss strategies for building partnerships and collaborations with stakeholders in the public, private, and nonprofit sectors.
Public Health is Glocal	Teaches about the interconnectedness of global and local factors in shaping public health outcomes and interventions. Through this module, students will examine how global health issues, policies, and trends impact local communities, as well as the role of local contexts, cultures, and systems in influencing global health initiatives.	Understand the concept of glocalization and its relevance to public health practice, policy, and research. Analyze the role of global health governance structures, international organizations, and partnerships in addressing public health challenges. Gain insights into the importance of community engagement, cultural competence, and contextual adaptation in global health programming.
Case Studies in Public Health	Provides students with opportunities to analyze real-world public health issues, interventions, and policies through case study analysis. Through this module, students will examine diverse public health challenges and responses, develop critical thinking skills, and apply theoretical knowledge to practical scenarios.	Understand the value of case study methodology in exploring complex public health issues and solutions. Analyze case studies of public health emergencies, disease outbreaks, health promotion campaigns, and policy interventions from diverse geographic, cultural, and socio-economic contexts. Identify key stakeholders, factors, and determinants influencing the outcomes of public health case studies. Examine the evidence base, theoretical frameworks, and best practices underlying successful public health interventions shown in case studies.

**Figure 1:**
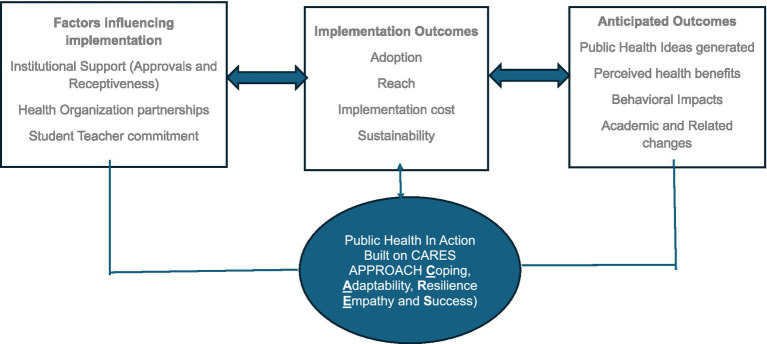
Model of public health in action in high schools.

This model can create triple benefits of being an educational institution, a well-being hub connecting young people to services, and as an enabler for behavior and health-related engagement (Figure 1).

### Expected student outcomes through this initiative

We employed multiple approaches to measure implementation and other anticipated outcomes through a series of instruments including self-report surveys, and observation. Some of the broad student outcomes expected include.

- Acquire a comprehensive understanding of key concepts, theories, and practices in public health.
- Develop critical thinking, analytical, communication, and teamwork skills essential for addressing public health challenges and contributing effectively to interdisciplinary teams.
- Make a positive impact on public health outcomes, whether through future academic pursuits, professional endeavors, or community engagement initiatives.
- Apply theoretical knowledge to practical situations through case studies, projects, and hands-on activities
- Learn how to present information, knowledge, and projects to a group

Further there are implementation and potential outcomes of the model and have been categorized as below.

- Implementation outcomes

Some of the metrics of implementation include.

- *Adoption:* The number and proportion of schools that initially decided to employ Public Health in Action curriculum.
- *Reach*: Refers to the number of students who are receiving Public Health in Action curriculum.
- *Implementation cost:* It is a quantified measure that assesses the financial impact of Public Health in Action curriculum.
- S*ustainability:* It is the scope in which the Public Health in Action curriculum is embedded within the school’s daily system.

- Anticipated outcomes

Some of the metrics of the anticipated outcomes include.

- *Student participation in public health clubs:* We track the number of students participating in the public health clubs.
- *Public Health Ideas generated:* High school students participate in an annual public health hackathon to present with innovative out of box ideas to address public health challenges impacting Memphis and beyond in the 21st century. We track the number of ideas generated and implemented.
- *Celebration of public health days:* We track if the public health days were celebrated by students in the high schools where the public health clubs have been established.
- *Students deciding to pursue public health careers:* We also track the number of students who decide to pursue public health careers either through public health dual enrollment, or interdisciplinary public health education or undergraduate education in public health.
- *Perceived health benefits:* Perception about stress, sleep, physical activity, sedentary behavior, meal habits were also assessed. Specifically, students engage with these topics through assignments such as problem identification, data exploration, needs assessment, and solution design related to stress, sleep patterns, and sedentary lifestyles.
- *Additional assessment on academic outcomes and related changes were made.*

### Implementation of RE-AIM public health IDEAS club in high schools

We plan to scale the implementation using the Context and Implementation of Complex Interventions (CICI) framework where Context comprises seven domains (i.e., geographical, epidemiological, socio-cultural, socio-economic, ethical, legal, political); implementation consists of five domains (i.e., implementation theory, process, strategies, agents and outcomes); setting refers to the specific physical location, in which the intervention is put into practice (19). The CICI framework comprises of three dimensions: context, implementation and setting interacting with one another and with the intervention dimension (19).

The public health clubs have been established across many high schools in US and India, and we continue to further expand these clubs in other countries. The curriculum Public Health In Action provides a structured foundation for each Public Health Club, but it is also flexible and adaptable. Clubs add fun activities, team-building exercises, and icebreakers as needed to keep members engaged. The goal is to ensure students are learning, collaborating, and creating meaningful public health solutions and building community while enjoying the process. The Public Health clubs are delivering the curriculum by a team of experts in public health including undergraduate and graduate students of the school of public health.

The 1st Public Health Club in high school activities began in February 2023. Few of the first few schools in our pilot included Medical District High School and University High School in Memphis. The initial implementation involved visiting the schools, meeting with high school students, orienting them to the field of public health, responding to their questions, giving examples of how public health is so important and how it influences individuals’ own health and the health of their families and the communities they live in. At the same time, students are provided with the public health career opportunities and what educational pathways are available for them to have a career in public health. These initial conversations and dialogue allowed us to gather feedback around youth perception about public health and was instrumental in framing our activities accordingly. One of the key aspects of the public health clubs was to visit high schools as desired by the school authorities so that there is consistency and continuity in the activities planned. This was important to build trust with the student community about this being not one time thing but an ongoing discussion and dialogue about public health and its importance. Since then, the clubs have been expanded to additional high schools in Memphis including Central High School, Cordova Junior and High School, Houston High School, Kingsbury High School, Southwind High School, Craigmont High School, Hamilton High School, Raleigh Egypt High School and Trezevant High School.

The University of Memphis School of Public Health signed memorandum of understanding with DAV University, India with one of the key components of this collaboration was to establish public health clubs in the high schools. DAV University, in collaboration with the University of Memphis School of Public Health, was granted a prestigious project by the Punjab Council of Science and Technology and Department of Science and Technology, Government of India aimed at establishing Public Health Clubs in schools across Jalandhar. Under this collaboration, Public Health Clubs have now been established across 10 Government Senior Secondary Schools (GSSS) in Jalandhar in the state of Punjab. These include GSSS Randhawa Masanda (established on August 4, 2025), GSSS Nurpur (August 5, 2025), GSSS Karari (August 5, 2025), GSSS Beas Pind (August 7, 2025), GSSS (Girls) Alwalpur (August 8, 2025), GSSS Kala Bakra (August 8, 2025), GSSS (Girls) Bhogpur (August 12, 2025), GSSS Binpalke (August 12, 2025), GHS Giganwal (August 13, 2025), and GSSS Pachranga (August 14, 2025). Other countries we are exploring to establish these public health clubs include including Portugal, Malta, Saudi Arabia and Eastern Central and Southern Africa.

### Public health club events and activities

Some planned events across public health clubs include celebrating World Food Day, World Diabetes Day, World AIDS Day, International Day of Persons with Disabilities, World Soil Day and Universal Health Coverage Day.

The activities focused around Sustainable Development Goals (20) including No Poverty (SDG1), Zero Hunger (SDG2), Good Health and Well-Being (SDG3), Quality Education (SDG4), Gender equality (SDG5), Clean water and sanitation (SDG6), Decent work and economic growth (SDG8), Industry, Innovation and Infrastructure (SDG9), Reduced Inequalities (SDG10), Sustainable Cities and Communities (SDG11), and Partnership for the Goals (SDG17).

This initiative is designed to promote public health education and empower students with practical skills and leadership qualities to tackle real-world health challenges. Through this program, students actively engage in improving community health by collaborating with peers from around the world. The focus areas of the initiative include hygiene, nutrition, mental well-being, and preventive healthcare. As part of the program, Public Health Clubs established in schools engage students in the following key activities:

- Celebrating Public Health Days Every Month: Each month, Public Health Clubs observe Public Health Day, highlighting different health themes to raise awareness, promote healthy lifestyles, and encourage students to take part in community health improvement activities.
- Public Health Workshops: The clubs conduct regular workshops to educate students and their families on critical public health topics, including hygiene, nutrition, and mental well-being.
- Community-Based Participatory Research: Students take part in research activities aimed at generating data and meaningful insights on pressing public health challenges, utilizing informatics and analytic skills.
- Public Health Campaigns: Evidence-based public health campaigns are created and conducted within the community to raise awareness and provide practical solutions to local health concerns.
- Participation in RE-AIM Public Health IDEAS Hackathons: Students engage in global hackathons through the RE-AIM Public Health IDEAS framework, allowing them to explore innovative solutions to community health issues.

### Example of the activities include

On May 23, 2024, we launched our first international public health club in a high school in India at the SDRV convent school, Greater Noida, India. Several activities included Cleanliness drive, celebrating World Heart Day, dengue awareness drive, awareness around sustainable development goals, mobile addiction, plantation drive, and world water day. In addition, the students also had an opportunity to participate in the prestigious International Conference, organized by the World Health Organization (WHO) in collaboration with the World Federation of United Nations Associations (WFUNA).

On October 02, 2024, at Kingsbury High, the activity titled *Introduction to Public Health* was conducted. Students participated in an initial hackathon promotion and club introduction, learning the foundational concepts of public health. The activity also helped build awareness about broader substance abuse prevention themes while engaging over 20 students.

On November 20, 2024, at Cordova High (Junior level), the activity titled *Community Health Issues - Systems Thinking and Problem Identification* was conducted. Students collaboratively identified local health problems to research and use as the foundation for future project designs.

On November 06, 2024, at Kingsbury High, the activity titled *Campaign Development* was conducted. The session involved poster design, data gathering, and presentations, with students assigned roles to develop modules addressing substance use prevention. Through design thinking sessions, students brainstormed preventive solutions for middle school audiences. They identified behavioral risks and crafted tailored interventions, linking public health innovation with real-world community needs.

**Figure 2:**
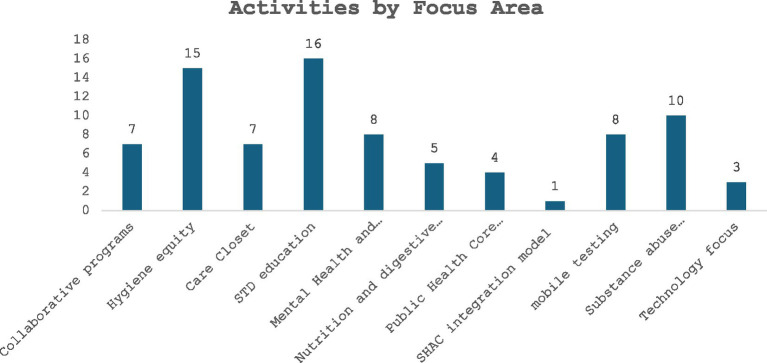
Number of activities by their focus areas.

The analysis of activities by focus area highlights both the scale and relative emphasis of different themes. For instance, Hygiene equity represented 15 activities (22%), while initiatives focused on collaborative programs accounted for 7 activities (10%) as well. A smaller cluster of 1–2 activities each (about 4–8%) addressed more specific or emerging priorities. The activity domains shown in Figure 2 reflect three broader conceptual clusters: (1) health behavior and condition-focused topics (e.g., hygiene equity, nutrition and digestive health, mental health, infectious disease, substance abuse prevention, STD education); (2) public health systems and core concepts (e.g., Public Health Core Concepts, SHAC integration model, mobile testing); and (3) programmatic and instructional approaches (e.g., collaborative programs, care closet initiatives, technology-focused activities). This organization highlights the diversity of student engagement while maintaining consistency in classification. This distribution shows that while hygiene-related initiatives and collaboration dominate the agenda, there is also meaningful attention given to targeted issues, ensuring a balance between broad structural challenges and specialized health concerns.

**Figure 3:**
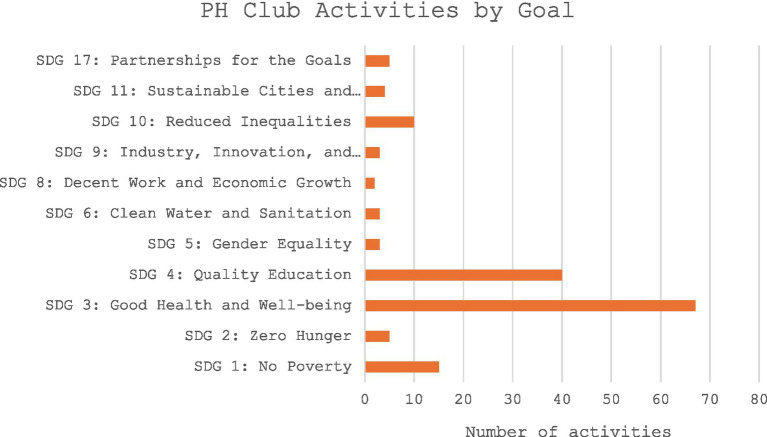
Public health club activities by SDG goals.

The Public Health Club activities by SDG goals reveal that most initiatives are strongly aligned with SDG 3: Good Health and Well-being, which accounted for 67 activities (42.7%), highlighting the central mission of promoting health. The second largest share was SDG 4: Quality Education with 40 activities (25.5%), reflecting the clubs’ role in raising awareness and building knowledge. Other notable contributions include SDG 1: No Poverty (15 activities, 9.6%) and SDG 10: Reduced Inequalities (10 activities, 6.4%), showing efforts to address broader social determinants of health. Smaller but meaningful activities supported SDG 2: Zero Hunger (5 activities, 3.2%), SDG 17: Partnerships for the Goals (5 activities, 3.2%), and others such as gender equality, clean water, innovation, and sustainable communities. Public Health Club activities were mapped to the Sustainable Development Goals (SDGs) through descriptive review, with each activity assigned to the SDG most closely aligned with its primary focus. Although some activities addressed multiple SDGs, classification reflects the dominant theme emphasized in student discussions, worksheets, and project outputs; the observed distribution highlights student-driven priorities within specific school and community contexts. This distribution underscores how the clubs not only prioritize health directly but also connect their work to education, equity, and sustainability (Figure 3).

Table 2 shows how Public Health Club activities were distributed across different schools. Cordova High (Senior) led with 15 activities involving 15 students, while Cordova High (Junior) followed with 14 activities engaging 35 students. Kingsbury High conducted 10 activities with 21 students, whereas Central High hosted 8 activities with 10 students. Southwind High also carried out 8 activities, engaging 87 students, marking the highest unique student participation despite having fewer activities. This distribution highlights that while Cordova High campuses led in activity count, Southwind High demonstrated the broadest student reach, showing different strengths across schools in mobilizing public health engagement.

**Table 2:** Summary table of PH club activities by school.

SN	School	Total activities	Total students
1	Cordova High (S)	15	15
2	Cordova High (J)	14	35
3	Kingsbury High	10	21
4	Central High	8	10
5	Southwind High	8	87
6	Multiple Schools	5	87
7	Trezevant CTC	5	12
8	All Hackathon Teams	1	87
9	Cordova High (J) and (S)	1	35
10	Houston High	1	Not specified

## Discussion

Schools have more influence on the lives of young people than any other social institution except the family. They provide a setting in which friendship networks develop, socialization occurs, and behavioral norms are developed and reinforced (21). Preventable health risk behaviors are often formed in childhood, persist into adulthood and are frequently interrelated (22). There has been significant progress in assessing the effectiveness of school interventions (23–25), and factors affecting implementation (26–28). However, there is less evidence about sustaining health interventions in schools beyond initial pilots. If effective interventions discontinue, new practices cannot reach wider populations and investments in time, people and resources to initiate and implement them may be wasted (29–32).

The establishment of Public Health Clubs in schools marks a significant step in empowering students to take charge of their health and well-being. By equipping them with essential knowledge and skills, this initiative will enable young minds to make a tangible difference in their own health and of their families and the communities they live in or beyond. Students who participate in public health education classes that use effective curricula have been found to increase their health knowledge and improve their health skills and behaviors. Teachers may need more support and preparation time to deliver curriculums that include health (33). The findings from this descriptive evaluation highlight how the *Re-AIM Public Health IDEAS* model effectively supports students in generating innovative, community-centered public health solutions, an outcome that can be further understood through the lens of the GITT (6). As students engaged in the *Public Health in Action* curriculum and Public Health Club activities, they interacted with diverse “information granules,” including public health concepts, real-world data, peer discussion, community observations, and lived experiences (14, 18, 34). According to GITT, such interactions enable individuals to absorb, filter, and reorganize information into meaningful mental products, such as creative ideas, problem-solving strategies, and values. The diversity of student project topics and their alignment with Sustainable Development Goals suggest that students were engaging with a range of ideas and applying concepts to community contexts.

Providing public health education opportunities in high school familiarizes students with relevant health information, expands opportunities to learn and practice organizational, leadership, and problem-solving skills, and normalizes positive health behaviors that could impact personal and community health outcomes for years to come (18, 35). Through this initiative, youth will be encouraged to pursue careers in public health, or even a career in health profession, enhance their sensitivity to health issues, and become public health ambassadors committed to translating education into impactful community solutions, thus countering the ongoing shrinkage of the number of health graduates in developed countries. The program also aims to improve overall community well-being by fostering a culture of proactive health awareness and care.

This initiative has several strengths. Introducing the field of public health education early in high schools allowed opportunity to understand youth perspective about public health through their lens, provided insights on the careers that public health offers, raised youth’s awareness about how innovative public health approaches can address the social, economic and health challenges. Another strength of this initiative is providing high school youth an opportunity to pursue public health as a career through public health dual enrollment and contribute to increasing community awareness about public health through public health clubs and ideate and innovate to address public health challenges through participation in public health hackathons. This innovative multi-prong approach to bringing public health education to high schools across local and global settings provides youth an opportunity to understand the importance of data driven evidence-based information for informed decision-making for greater community impact.

There are some limitations of the study. One of the limitations is tracking in a formal objective way of how the model of public health education in high schools is enhancing good health and well-being of the youth, their families and the communities they live in. The evaluation framework is currently in progress and the findings will be reported in near future. The focus of this study was to report on the feasibility of the implementation of the model of public health education in the high schools. Another key limitation is demographic characteristics of individual students were not systematically collected, as the initiative was implemented as a pilot educational program rather than a research study. This limits the ability to assess participant representativeness or examine differential outcomes across subgroups. Future research should include demographic data collection to enhance interpretability and generalizability.

### Implications

#### Theoretical implications

This study contributes to theoretical discussions in public health education by illustrating how structured, school-based programs can operationalize public health concepts for adolescent learners. The Re-AIM Public Health IDEAS model extends existing frameworks by applying them specifically to high school students and by integrating entrepreneurial and innovation-oriented elements through the RE-AIM components (Research, Entrepreneurship, Analytics, Informatics, and Management) alongside the CARES pillars. This approach promotes systems thinking, creativity, and engagement, demonstrating how experiential and interdisciplinary learning environments can support youth in synthesizing information, identifying community health challenges, and generating innovative, contextually grounded solutions.

#### Practical implications

From a practice perspective, the findings suggest that school-based public health initiatives can serve as effective platforms for engaging students beyond traditional classroom instruction. Educators and school leaders may use the Re-AIM Public Health IDEAS model to strengthen extracurricular programming, foster interdisciplinary collaboration, and create pathways for youth participation in community health activities. The model offers a flexible structure that can be adapted across diverse school settings and aligned with existing educational goals.

Implications for Future Research: Future research should build on this pilot work by employing prospective study designs, comparison groups, and validated outcome measures to more rigorously assess program effectiveness. Longitudinal studies could examine sustained impacts on students’ academic trajectories, public health competencies, and engagement. Additionally, research across varied geographic and institutional contexts would strengthen generalizability and address limitations related to sample size and setting.

### Policy recommendations

To advance the integration of public health education within high school settings, several policy actions are recommended. First, education systems should consider embedding foundational public health concepts into secondary school curricula, using experiential and project-based approaches that encourage student creativity and problem solving. Second, schools should be supported in formally establishing Public Health Clubs as co-curricular platforms where students can collaborate, develop leadership skills, and address community health challenges. Third, policymakers should invest in training and professional development for teachers so they can confidently guide students through public health topics and project-based learning. Finally, partnerships between schools, universities, public health agencies, and global organizations should be expanded to provide mentorship, resources, and opportunities for youth-led initiatives. These efforts can help build a generation of students equipped with the skills and motivation to engage in public health action locally and globally.

## Conclusion

The *RE-AIM Public Health IDEAS* model and the *Public Health in Action* curriculum provide a scalable and adaptable framework for integrating public health education into high schools. The implementation of the *Re-AIM Public Health IDEAS* model across more than 20 national and international schools demonstrates the feasibility and global relevance of school-based public health education. With nearly 400 students participating in more than 150 activities aligned with the Sustainable Development Goals, the initiative fostered collaboration, creativity, and community engagement among youth. By focusing on hygiene, nutrition, mental well-being, and preventive healthcare, the program showed strong potential to empower students as emerging public health leaders committed to improving community health locally and globally.

By equipping students with critical thinking, problem-solving, and leadership skills, and by fostering youth engagement through Public Health Clubs, the initiative demonstrates the potential to cultivate a new generation of public health ambassadors. This approach not only empowers adolescents to address pressing community health challenges but also contributes to building healthier, more resilient societies. Expanding such programs globally can support Sustainable Development Goals by promoting inclusivity, well-being, and social responsibility among youth. Sustainability and the long-term impact of this model on student career pathways, community health outcomes, and policy integration, may reinforce it as an innovative strategy in public health education.

## Data Availability

The original contributions presented in the study are included in the article/supplementary material, further inquiries can be directed to the corresponding author.

## References

[1] Pulimeno M Piscitelli P Colazzo S Colao A Miani A. School as ideal setting to promote health and wellbeing among young people. Health Promot Perspect. (2020) 10:316–24. doi: 10.34172/hpp.2020.50, 33312927 PMC7723000

[2] United Nations. Unsdg | TOGETHER POSSIBLE: Gearing up for the 2030 Agenda. (2025). Available online at: https://unsdg.un.org/resources/together-possible-gearing-2030-agenda (Accessed September 29, 2025)

[3] WHO. (2025). Mental health of adolescents [Internet]. Available online at: https://www.who.int/news-room/fact-sheets/detail/adolescent-mental-health (Accessed March 16, 2026)

[4] Bawden A. Half a billion young people will be obese or overweight by 2030, report finds. The Guardian. (2025). Available online at: https://www.theguardian.com/society/2025/may/20/young-people-obesity-2030-report (Accessed March 16, 2026)

[5] Adams K Monahan J Wills R. Losing the whole child? A national survey of primary education training provision for spiritual, moral, social and cultural development. Eur J Teach Educ. (2015) 38:199–216. doi: 10.1080/02619768.2015.1030388

[6] Nguyen MH Jin R Hoang G Nguyen MH Nguyen L Le T . Examining contributors to Vietnamese high school students’ digital creativity under the serendipity-mindsponge-3D knowledge management framework. Think Skills Creat. (2023) 49:101350. doi: 10.1016/j.tsc.2023.101350

[7] Darling-Hammond L. Constructing 21st-century teacher education. J Teach Educ. (2006) 57:300–14. doi: 10.1177/0022487105285962

[8] Peterson FL Cooper RJ Laird JA t. Enhancing teacher health literacy in school health promotion a vision for the new millennium. J Sch Health. (2001) 71:138–44. doi: 10.1111/j.1746-1561.2001.tb01311.x, 11354982

[9] Vega V. Robb MB. The Common Sense Census: Inside the 21st-Century Classroom | Common Sense Media. (2019). Report No. Available online at: https://www.commonsensemedia.org/research/the-common-sense-census-inside-the-21st-century-classroom (Accessed October 26, 2025)

[10] Yoon S An S Noh DH Tuan LT Lee J. Effects of health education on adolescents’ non-cognitive skills, life satisfaction and aspirations, and health-related quality of life: a cluster-randomized controlled trial in Vietnam. PLoS One. (2021) 16:e0259000. doi: 10.1371/journal.pone.0259000, 34851980 PMC8635366

[11] Vuong Q Nguyen MH. Exploring the role of rejection in scholarly knowledge production: insights from granular interaction thinking and information theory. Learned Publ. (2024) 37:e1636. doi: 10.1002/leap.1636

[12] Mersal FA Alenezi IN. Gender-specific pathways to leadership competency: the role of emotional intelligence and self-esteem among Saudi nursing students. Front Med. (2026) 12:12. doi: 10.3389/fmed.2025.1744198, 41601747 PMC12832521

[13] Duong MPT Nguyen MH Sari NPWP Nguyen HHT Vuong QH. Urban residents’ willingness to finance public park tree-planting: the role of biodiversity loss perceptions and park visits. Sustainability. (2026) 18:2706. doi: 10.3390/su18062706

[14] University of Memphis. Public Health in Action Initiative - School of Public Health. (2025). Available online at: https://www.memphis.edu/publichealth/initiatives/ph-in-action/about-phia.php (Accessed December 9, 2025)

[15] Rose CD. Ethical conduct of research in children: pediatricians and their IRB (part 1 of 2). Pediatrics. (2017) 139:e20163648. doi: 10.1542/peds.2016-3648, 28557746

[16] Federal Register. (2015). Federal Policy for the Protection of Human Subjects. Available online at: https://www.federalregister.gov/documents/2015/09/08/2015-21756/federal-policy-for-the-protection-of-human-subjects (Accessed March 16, 2026)

[17] University of Memphis. RE-AIM Public Health IDEAS Club: Activities. (2025). Available online at: https://www.memphis.edu/publichealth/initiatives/ph-clubs/activities.php (Accessed September 29, 2025)

[18] Joshi A Jha N Taylor M Saulsberry-Scarboro N Orians C Parish S . The public health CARES initiative: a framework for public health education in high schools using the SMAART model. Front Public Health. (2025) 13:1563971. doi: 10.3389/fpubh.2025.1563971, 40709035 PMC12286929

[19] Pfadenhauer LM Gerhardus A Mozygemba K Lysdahl KB Booth A Hofmann B . Making sense of complexity in context and implementation: the context and implementation of complex interventions (CICI) framework. Implement Sci. (2017) 12:21. doi: 10.1186/s13012-017-0552-5, 28202031 PMC5312531

[20] UInited Nations. THE 17 GOALS | Sustainable Development. Available online at: https://sdgs.un.org/goals (Accessed September 29, 2025)

[21] UNESCO. Education 2030: Incheon Declaration and Framework for Action for the Implementation of Sustainable Development Goal 4. (2025) Available online at: https://unesdoc.unesco.org/ark:/48223/pf0000245656/Pdf/245656eng.pdf.multi (Accessed September 29, 2025)

[22] Stewart-Brown S. WHO Regional Office for Europe (Health Evidence Network Report). What is the Evidence on School Health Promotion in Improving Health or Preventing Disease and, Specifically, What is the Effectiveness of the Health Promoting Schools Approach | Mind Health. (2025). Available online at: http://www.mentalhealthpromotion.net/?i=training.en.bibliography.1141 (Accessed September 29, 2025)

[23] Shackleton N Jamal F Viner RM Dickson K Patton G Bonell C. School-based interventions going beyond health education to promote adolescent health: systematic review of reviews. J Adolesc Health Off Publ Soc Adolesc Med. (2016) 58:382–96. doi: 10.1016/j.jadohealth.2015.12.017, 27013271

[24] Langford R Bonell CP Jones HE Pouliou T Murphy SM Waters E (2014). The WHO health promoting school framework for improving the health and well-being of students and their academic achievement - Langford, R - 2014 | Cochrane library. Available online at: https://www.cochranelibrary.com/cdsr/doi/10.1002/14651858.CD008958.pub2/full (Accessed September 29, 2025) 10.1002/14651858.CD008958.pub2 PMC11214127 24737131

[25] Bonell C Jamal F Harden A Wells H Parry W Fletcher A . Systematic review of the effects of schools and school environment interventions on health: evidence mapping and synthesis. Public Health Res. (2013) 1:1–320. doi: 10.3310/phr01010 25642578

[26] Domitrovich CE Bradshaw CP Poduska JM Hoagwood K Buckley JA Olin S . Maximizing the implementation quality of evidence-based preventive interventions in schools: a conceptual framework. Adv Sch Ment Health Promot. (2008) 1:6–28. doi: 10.1080/1754730x.2008.9715730, 27182282 PMC4865398

[27] Pearson M Chilton R Wyatt K Abraham C Ford T Woods H . Implementing health promotion programmes in schools: a realist systematic review of research and experience in the United Kingdom. Implement Sci. (2015) 10:149. doi: 10.1186/s13012-015-0338-6, 26510493 PMC4625879

[28] Darlington EJ Violon N Jourdan D. Implementation of health promotion programmes in schools: an approach to understand the influence of contextual factors on the process? BMC Public Health. (2018) 18:163. doi: 10.1186/s12889-017-5011-3, 29357922 PMC5776776

[29] Schell SF Luke DA Schooley MW Elliott MB Herbers SH Mueller NB . Public health program capacity for sustainability: a new framework. Implement Sci. (2013) 8:15. doi: 10.1186/1748-5908-8-15, 23375082 PMC3599102

[30] Bumbarger B Perkins D. After randomised trials: issues related to dissemination of evidence-based interventions. J Child Serv. (2008) 3:55–64. doi: 10.1108/17466660200800012

[31] Scheirer MA Dearing JW. An agenda for research on the sustainability of public health programs. Am J Public Health. (2011) 101:2059–67. doi: 10.2105/AJPH.2011.300193, 21940916 PMC3222409

[32] Glasgow RE Vogt TM Boles SM. Evaluating the public health impact of health promotion interventions: the RE-AIM framework. Am J Public Health. (1999) 89:1322–7. doi: 10.2105/ajph.89.9.1322, 10474547 PMC1508772

[33] Tancred T Paparini S Melendez-Torres GJ Fletcher A Thomas J Campbell R . Interventions integrating health and academic interventions to prevent substance use and violence: a systematic review and synthesis of process evaluations. Syst Rev. (2018) 7:227. doi: 10.1186/s13643-018-0886-3, 30522529 PMC6284294

[34] Vuong QH La VP Nguyen MH. Informational entropy-based value formation: a new paradigm for a deeper understanding of value. Eval Rev. (2025):193841X251396210. doi: 10.1177/0193841X251396210, 41214459

[35] University of Memphis. A Model for Public Health Education in High Schools. Available online at: https://www.memphis.edu/publichealth/initiatives/reaim/reaim-about.php (Accessed September 29, 2025)

